# Perspectives for cancer immunotherapy mediated by p19Arf plus interferon-beta gene transfer

**DOI:** 10.6061/clinics/2018/e479s

**Published:** 2018-08-28

**Authors:** Bryan E Strauss, Gissele Rolemberg Oliveira Silva, Igor de Luna Vieira, Otto Luiz Dutra Cerqueira, Paulo Roberto Del Valle, Ruan Felipe Vieira Medrano, Samir Andrade Mendonça

**Affiliations:** Laboratório de Vetores Virais, Centro de Investigação Translacional em Oncologia, Instituto do Cancer do Estado de Sao Paulo (ICESP), Hospital das Clinicas HCFMUSP, Faculdade de Medicina, Universidade de Sao Paulo, Sao Paulo, SP, BR

**Keywords:** Melanoma, Immunotherapy, Immunogenic Cell Death, Translational Medicine, Viral Vectors

## Abstract

While cancer immunotherapy has gained much deserved attention in recent years, many areas regarding the optimization of such modalities remain unexplored, including the development of novel approaches and the strategic combination of therapies that target multiple aspects of the cancer-immunity cycle. Our own work involves the use of gene transfer technology to promote cell death and immune stimulation. Such immunogenic cell death, mediated by the combined transfer of the alternate reading frame (p14ARF in humans and p19Arf in mice) and the interferon-β cDNA in our case, was shown to promote an antitumor immune response in mouse models of melanoma and lung carcinoma. With these encouraging results, we are now setting out on the road toward translational and preclinical development of our novel immunotherapeutic approach. Here, we outline the perspectives and challenges that we face, including the use of human tumor and immune cells to verify the response seen in mouse models and the incorporation of clinically relevant models, such as patient-derived xenografts and spontaneous tumors in animals. In addition, we seek to combine our immunotherapeutic approach with other treatments, such as chemotherapy or checkpoint blockade, with the goal of reducing dosage and increasing efficacy. The success of any translational research requires the cooperation of a multidisciplinary team of professionals involved in laboratory and clinical research, a relationship that is fostered at the Cancer Institute of Sao Paulo.

## INTRODUCTION

The progression of cancers depends, in part, on the ability of tumor cells to escape immunosurveillance 1. Tumor cells accomplish this by a variety of mechanisms, collectively termed immunoediting, which include the hijacking of signaling events to promote an immunosuppressive microenvironment and selecting tumor cells that are no longer recognized by the immune system 2,3. The goal of immunotherapy is to enhance, if not reboot, the cancer-immunity cycle, starting with tumor cell killing by an immunogenic mechanism, enhancing the function of antigen-presenting cells (APCs) and stimulating cytolytic and helper T (Th) cell responses, thus completing the cycle 4. Each round through the immunity cycle may amplify the antitumor immune response since the killing of the tumor cells may release additional antigens that would then contribute to broaden the T cell repertoire.

Immunotherapies that directly target tumor cell killing typically rely on the induction of immunogenic cell death (ICD). Such therapeutic approaches include treatment with certain chemotherapeutic agents, such as anthracyclines and oxaliplatin, or the application of oncolytic viruses. These agents promote the release of tumor antigens in conjunction with the release of signals that activate APCs, including ATP, high mobility group box-1 (HMGB1) and interferon-β (IFNβ), and the exposure of calreticulin on the surface of tumor cells 5. Alternatively, tumor vaccines may provide one or more critical antigens and may even deliver *ex vivo*-modified dendritic cells (DCs) to stimulate an antitumor immune response. Checkpoint blockade typically relies on the use of monoclonal antibodies to reverse the negative regulation of T cells due to their expression of inhibitory molecules, such as cytotoxic T lymphocyte-associated molecule-4 (CTLA-4) or programmed cell death protein 1 (PD-1) 4. Alternatively, adoptive cell transfer (ACT) can be used, where autologous T cells may be selected/modified *ex vivo*, expanded, and returned to the patient to induce tumor cell killing. In particular, chimeric antigen receptor (CAR) T cells have recently gained much attention, especially for the treatment of B cell leukemias 6 and lymphomas (https://www.fda.gov/NewsEvents/Newsroom/PressAnnouncements/ucm 581216.htm). Since each of these immunotherapies targets particular points in the cancer-immunity cycle, their combination may also lead to an even greater efficacy 4.

The promise of immunotherapies has justifiably gained considerable attention over the past few years. However, the role of gene transfer/gene therapy in this setting has not yet been fully explored. For example, oncolytic viruses induce cell killing as a result of viral replication leading to cell lysis but do not necessarily encode a transgene, such as granulocyte macrophage-colony stimulating factor (GM-CSF). The application of oncolytic viruses is often referred to as virotherapy, a close cousin of gene therapy, but not a gene transfer approach per se. In fact, Imlygic (talimogene laherparepvec, T-Vec, an oncolytic herpes virus encoding GM-CSF) was considered a first in-class product when it was approved by the U.S. Food and Drug Administration (FDA) 7. The recently approved CAR T cell approaches Kymriah and Yescarta for the treatment of B cell leukemia and some large B cell lymphomas, respectively 6, (http://www.fda.gov/NewsEvents/Newsroom/PressAnnouncements/ucm581216.htm) have been classified by the FDA as cell-based gene therapies; that is, the gene transfer aspect is not performed directly in the patient, but a vector encoding the CAR is applied to T cells *ex vivo*.

To the best of our knowledge, no *in situ* cancer gene therapy approach that acts as an inducer of ICD, characterized by the release of ATP, calreticulin and HMGB1, has been described to date. Nevertheless, gene therapy approaches that induce an immune response are known. The transfer of the thymidine kinase (TK) gene derived from the herpes simplex virus by means of nonreplicating adenoviral vectors (Ad-TK) has been extensively explored 8. In transduced tumor cells, TK, in conjunction with cellular enzymes, converts prodrugs (ganciclovir, valacyclovir, acyclovir) into their active forms to block DNA replication and induce cell death. The Ad-TK approach, also termed gene-mediated cytotoxic immunotherapy (GMCI), is known not only for the associated bystander effect but also for its ability to stimulate an antitumor immune response 8. Several clinical trials are being or have been performed, including a phase III trial for the treatment of high-grade glioma, where time to death, but not overall survival, was increased 9.

Also in development are approaches that combine GMCI with other therapeutics that boost the antitumor response, including the association of Ad-TK with FMS-like tyrosine kinase 3 ligand (FLT3L) gene transfer 10. A particularly interesting approach is the use of Toca 511 (vocimagene amiretrorepvec), a nonlytic, replicating retroviral vector that spreads among tumor cells for the delivery of the cytosine deaminase (CD) gene, which converts 5-fluorocytosine into 5-fluorouracil (5FU) and has been shown stimulate antitumor immune responses 11. In a phase I trial, compared to an external control, Toca 511 significantly improved the overall survival of patients with high-grade glioma 12. While a variety of gene transfer approaches can be considered immunotherapies, further improvements may result in more robust responses in a larger number of patients.

### A role for the p19Arf and IFNβ gene transfer in cancer immunotherapy

Our work has focused on cancer gene therapy using adenovirus-mediated gene transfer to elicit both cell death and activation of an immune response against tumors. Here, we will provide an overview of one immunotherapeutic approach that utilizes a specialized vector to deliver the cDNA encoding the alternate reading frame (ARF; p14ARF in humans and p19Arf in mice) and IFNβ proteins to cancer cells. Our group is also developing additional modalities described elsewhere, including a review in this issue of Clinics, which involve the use of our specialized vector for the transfer of the cDNA encoding the tumor suppressor p53. The following discussion will provide an overview of the development of our gene transfer approach and of the evidence suggesting that the transfer of p19Arf and IFNβ indeed acts as an immunotherapy in mouse models of melanoma and lung cancer.

The antitumor activities of p53 are frequently related to its role as a regulator of transcription of a variety of target genes, which in turn direct cell death, inhibit the cell cycle and DNA repair, and block angiogenesis, among others 13,14. Although quite complex, key regulators of p53 include the human homolog of murine double minute-2 (HDM2), which directs p53 for degradation, and p14ARF, which disrupts the interaction between MDM2 and p53, thus freeing p53 to act 15. Despite variable reports, published data indicate that up to 90% of melanomas retain p53 in the wild-type form 16. However, p53 is essentially dormant due to the loss of agonistic or gain in antagonistic factors, including the lack of p14ARF in 50% and the overexpression of HDM2 in 56% of melanomas 16-18. We reason that the endogenous wild-type p53 (p53wt) may be activated in response to gene transfer and that the activated p53, a powerful transcription factor, may be harnessed to not only act as a tumor suppressor but also drive the expression of the transgene encoded by the gene transfer vector as described below. Thus, we expect to establish dynamic interactions among the gene transfer vector, the therapeutic gene(s) and endogenous p53.

Our gene transfer platform involves a nonreplicating, recombinant, serotype 5 adenovirus (Ad5) in which transgene expression is controlled by a p53-responsive promoter, PGTxβ, called PG for simplicity. The chimeric promoter includes 13 copies of a p53-responsive element (PG), a TATA box (Tx) and the first intron of the rabbit β-globin gene (β), as previously detailed 19. The PG promoter can be used to drive the expression of the p53 cDNA, establishing a positive feedback mechanism that is initiated due to leaky transcription even in p53-null cells 20,21. Alternatively, the PG promoter may be employed to drive the transcription of any gene of choice as long as p53 is present in the cell. Expression from the PG promoter is 5 to 7 times higher than that seen from typical, constitutive promoters, such as the cytomegalovirus (CMV) immediate early promoter enhancer or the retroviral long terminal repeat (LTR) 19-21. In fact, we have developed three viral platforms, namely, retrovirus, adenovirus and adeno-associated virus, in which transgene expression is dependent on p53 19,22,23. Such vectors are expected to have utility in not only models of cancer gene therapy but also other conditions that involve cellular stress, such as hypoxia 22,24.

After developing the PG vectors, our next task was to use these to deliver the p19Arf cDNA and assess whether endogenous p53 could be activated. Indeed, the transfer of p19Arf, but not p53, resulted in increased p53 activity in B16 mouse melanoma cells (with endogenous p53wt). However, we noted that this activation was accentuated when gene transfer was combined with chemotherapeutic agents, resulting in increased cell death both *in vitro* and *in vivo* 25.

Since we wished to maximize cell death and induce immune stimulation, we decided to perform simultaneous transfer of p19Arf and IFNβ because the p53/Arf and type I interferon (IFNα/β) pathways have been indicated to cooperate. The presence of p53 has been shown to increase cell death and minimize viral replication in cells treated with recombinant type I interferon protein (rIFN) 26. Interestingly, one report form the literature suggests that ARF, not p53, is actually the critical factor that mediates the apoptotic response to rIFN 27. Such interactions are to be expected since both type I IFN and the p53/Arf pathways play important roles in regulating cell death 28. Indeed, we noted that compared to single gene transfer, the combined transfer of p19Arf and IFNβ (p19Arf + IFNβ) mediated by our p53-responsive Ad5 vector resulted in enhanced B16 cell death regardless of *in vitro* or *in vivo* application. In a mouse model of *in situ* gene therapy (that is, injection of viral particles directly into the tumor mass), only the combined gene transfer approach was associated with extensive tumor cell killing and prolonged survival, although IFNβ gene transfer could also delay tumor progression 29. From this study, we learned that the combined, but not individual, gene transfer was especially effective for the induction of cell death.

In a later study, we examined both the critical aspects of the adenovirus-mediated gene transfer and the specific mechanism of cell death in response to p19Arf and IFNβ gene transfer. In this work, improvements to vector design involved the use of the RGD tripeptide included in the knob domain of the adenovirus fiber protein, thus broadening the spectrum of cells that may be transduced since virus-cell interaction depends on integrins but not the coxsackievirus and adenovirus receptor 30-32. We also explored the use of a single vector for the simultaneous transfer of the p19Arf and IFNβ cDNA, finding that both co-transduction and IRES-mediated expression of two proteins from a single transcript were equally effective 32. As expected, combined, but not single, gene transfer resulted in enhanced killing of B16 cells. However, we uncovered an important bystander effect, where the presence of exogenous p19Arf was shown to sensitize the melanoma cells to secreted IFNβ produced by neighboring cells that had received the IFNβ vector. In addition, we provided functional and molecular evidence indicating that the presence of the adenoviral vector itself was important for mediating the antiviral response that contributed to cell killing. In other words, gene transfer was more effective than the pharmacological activation of the p53/Arf and type I IFN pathways 32.

The mechanism of cell death was also explored, revealing that apoptosis was not responsible for cell death in response to combined gene transfer, since caspase activity was not required for cell killing. Instead, we observed that combined, but not individual, gene transfer activated critical mediators of necroptosis. Moreover, only combined gene transfer was associated with the presentation of all three classic markers of ICD, namely, calreticulin, ATP and HMGB1. As expected, involvement of the p53 pathway was revealed by elevated levels of p53 target genes (p21/Cdkn2a, Puma, Phlda2), as well as p53 itself, when B16 cells were treated with combined gene transfer both *in vitro* and *in vivo*. Interestingly, combined gene transfer *in vivo* in a model of *in situ* gene therapy was associated with vesicular and perinuclear staining of LC3β, suggesting a potential role for autophagy in our model. Microarray analysis of B16 cells after *ex vivo* gene transfer revealed increased expression of genes associated with the immune response, p53 activity, cell death and antiviral response and decreased expression of cell cycle-related transcripts. Therefore, the multimodal cell death mechanism was consistent with necroptosis associated with the release of ICD markers and an antiviral response 31,32.

The cellular response to p19Arf + IFNβ gene transfer shows that our approach is effective at killing tumor cells by a mechanism that is expected to promote an antitumor immune response. In another study using the B16 mouse melanoma cell line, several parameters of this immune response were revealed. For example, B16 cells were transduced *ex vivo*, and then the dying cells were applied as a prophylactic vaccine in syngeneic immunocompetent mice. Later, naïve B16 cells were implanted in the opposite flank, representing a tumor challenge. While vaccinations involving IFNβ gene transfer alone or in combination with p19Arf were effective in slowing challenge tumor growth, only the combined approach was significantly associated with increased survival 33. With optimization, tumor progression at the vaccination site could be completely eliminated in both immunocompetent C57BL/6 or T cell-deficient BALB/c nude mice (both endowed with high activity of natural killer (NK) cells); however, this protection was lost in innate and adaptive immunodeficient NOD-SCID mice, suggesting a critical involvement of NK cells. Gene expression analysis revealed substantial upregulation of Ulbp1, IL-16, Killer/DR5 and FAS/Apo1, which are critical factors for the NK cell response, albeit only in association with the combined gene transfer approach. Evidence for a *de facto* immune response was revealed by the depletion of either CD4^+^ or CD8^+^ T cell populations, which reversed the protective effect of the prophylactic vaccine. Moreover, the vaccination approach promoted a Th1 cytokine profile. Strikingly, the optimized prophylactic vaccination protocol provided significant protection against tumor growth even when the challenge was applied 70 days postvaccination. Alternatively, subcutaneous Tm1 tumors were first established before the application of a therapeutic vaccine consisting of dying Tm1 cells in response to *ex vivo* gene transfer and resulted in a significant reduction of tumor progression only in response to the combined p19Arf + IFNβ treatment 33. In other words, our gene transfer approach with p19Arf + IFNβ can be classified as immunotherapy.

Although the vaccination approach involving p19Arf + IFNβ gene transfer provided evidence for an effective antitumor immune response, we also wished to explore whether *in situ* cancer gene therapy using our vectors can serve as immunotherapy. To this end, we first confirmed the induction of ICD in Lewis lung carcinoma (LLC, mouse lung carcinoma, p53wt) cells only in response to the combined gene transfer 34. We then showed that a prophylactic vaccination model using LLC cells in C57BL/6 mice was especially effective in slowing challenge tumor growth when the vaccine cells had been treated with the combination of p19Arf + IFNβ. *In situ* gene therapy showed that both IFNβ alone and in combination with p19Arf could significantly delay tumor progression, although the association of cisplatin with gene therapy resulted in nearly complete tumor inhibition only in the presence of p19Arf + IFNβ. Indeed, *in situ* gene therapy followed by subcutaneous injection of naïve LLC cells resulted in greatly enhanced inhibition of the challenge tumor when the primary tumor had been treated with p19Arf + IFNβ rather than IFNβ alone. In addition, microarray analysis of tumors treated with *in situ* gene therapy revealed a gene signature consistent with an immune response and chemotactic enrichment, suggesting the presence of neutrophils and CD8^+^ T cells. In one study, the presence of infiltrating neutrophils was confirmed, and depletion of the granulocyte population reversed the benefit of *in situ* gene therapy 34. In this study, we revealed, among other findings, that our *in situ* gene transfer approach initiates an antitumor immune response and can be considered immunotherapy.

Taken together, these results reveal that the p19Arf + IFNβ gene transfer strategy promotes an immune response that includes the participation of NK cells, neutrophils, a Th1 response and, importantly, CD4^+^ and CD8^+^ T cells 31,35. The cellular response to combined gene transfer has been shown to induce ICD and, specifically in the case of B16, cell death consistent with necroptosis 31,35. While the studies performed to date have been interesting, some developments remain before we can affirm that our approach indeed holds promise for clinical application.

## CHALLENGES AND PERSPECTIVES FOR THE P19ARF + IFN<$>{\TF="GREEK_B"\CHAR98} <$>β IMMUNOTHERAPY

**Figure 1:**
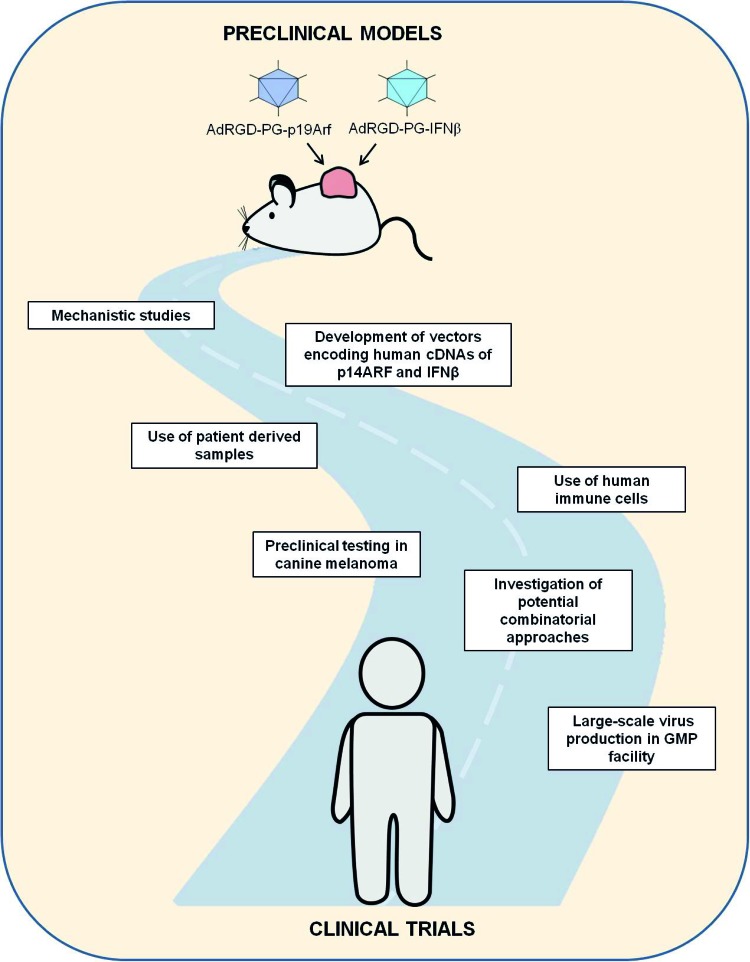
Schematic representation of the challenges and perspectives of continuing along the road of translational research. Critical milestones include testing our approach in human cells, including patient-derived tumor and immune cells, and models of spontaneous tumors, such as canine melanoma. In addition, the combination of our approach with chemotherapy or other immunotherapies may enhance efficacy. Virus production following good manufacturing practices (GMP) is critical for providing not only larger quantities of vectors but also biological agents of pharmaceutical quality.

As described in the following sections, we are looking forward to the next phase of our investigation where we face the challenge of further developing our approach and incorporating more clinically relevant models. In other words, we are poised to advance on our journey along translational research (Figure 1). We are concerned with the interactions of the p19Arf + IFNβ gene transfer approach with respect to not only the tumor cell response but also the impact on the tumor microenvironment. While the use of mouse models aids in such studies, especially when the immune response is to be examined, it is critical to determine the behavior of human tumor cells and even the activation of human immune cells upon p14ARF + human IFNβ (hIFNβ) gene transfer. Another critical step towards the preclinical evaluation of our immunotherapeutic approach involves the use of alternative models of tumor treatment.

### Potential impact of p19Arf + IFNβ on tumor angiogenesis

Since both the p53/Arf and IFN pathways are known to impact the tumor microenvironment, the study of such interactions may show the potential of our gene therapy approach in inhibiting angiogenesis and possibly metastasis. Tumor cells, nontumor cells, and noncellular components are partners working together for the survival of solid cancers 36. Nontumor cells are important components of the microenvironment, promoting tumorigenesis through a variety of cell types and mechanisms 37. The network of blood vessels is a critical component of the tumor microenvironment and provides oxygen, nutrients, immune surveillance and a route for metastasis, essentially fueling tumorigenesis 38. Failure of many cancer treatments can be explained, in part, by mechanisms that include poor biodistribution of drugs and an unfavorable tumor microenvironment 39. Thus, the impact of novel treatments on the tumor microenvironment must not be overlooked.

In addition to the well-known tumor suppressor activity of p53, its influence can reach beyond the single tumor cell 40. Depending on whether it is wild type or mutant, the p53 protein can modulate the extracellular matrix 41,42 and induce the secretion of proinflammatory proteins 43-45 and lactate, resulting in the acidification of the tumor boundary 46,47. p53 also coordinates the crosstalk between cancer and noncancer cells 48,49.

A p53-dependent and independent role for p19Arf in the angiogenic switch has been shown to accelerate tumor growth 50. Alternatively, p14ARF can sequester hypoxia-inducible factor 1α (HIF-1α), thus inhibiting HIF-1 transactivation in a p53-independent manner 51. Notably, p14ARF has been shown to suppress angiogenesis by blocking the translation of the vascular endothelial growth factor (VEGF)-A transcript 52. Thus, Arf can play a role in inhibiting tumor angiogenesis.

Type I IFNs are also recognized for their ability to block angiogenesis 28. For example, IFNβ has been shown to upregulate inducible nitric oxide synthase (iNOS), thus blocking angiogenesis and tumor progression in a xenograft model of human prostate carcinoma 53. IFNα and IFNβ have also been shown to block the production of essential mediators of angiogenesis, including basic fibroblast growth factor (bFGF), VEGF and interleukin (IL)-8 28. Thus, each of the central players in our gene transfer approach may negatively impact tumor angiogenesis, but this point remains to be experimentally tested.

### Gearing up for the study of p14ARF + hIFNβ gene transfer in human cells

Despite good results in preclinical models, many promising candidate drugs do not successfully cross the bridge between the bench and bedside 54. As discussed above, the combined p19Arf + IFNβ gene transfer approach triggered cell death mechanisms with remarkable immunogenic features, leading to reduced tumor burden and increased survival in animal models, which are desirable characteristics for any cancer therapeutics in development. Although our gene transfer approach seems promising, the translation of these results from mouse models to human cells represents a major step forward toward clinical relevance.

Mice and humans share many functional gene sequences but greatly diverge with respect to transcriptional regulation 55. Reliance on mouse models to predict human physiology and response to therapy is severely limited, and a critical step in closing this gap is the inclusion of studies performed in human cells. While patient-derived xenograft (PDX) models may be preferred 56, the use of established cell lines comes with ease of use and a wealth of knowledge in the literature about their genotype and response to various treatments. For example, a variety of human melanoma cell lines with distinct genotypes, especially with respect to p53, are widely available.

Nonetheless, the transition to human cell lines comes with an additional task with respect to our gene transfer approach. The cellular response to IFNβ is species specific; that is, mouse cells treated with human IFNβ, or vice versa, will produce distinct antiproliferative responses 57. Thus, we have to be particularly careful to match the cDNA encoded in the vectors with the species being treated. The assays described for B16 and LLC cells above used only mouse cDNA. To initiate tests in human cells, a new set of vectors harboring human protein coding sequences must be constructed. Human cDNA (p14ARF and hIFNβ) encoded by the p53-responsive adenoviral vectors and applied to human melanoma cell lines creates the opportunity to test the induction of cell death, cooperation between the p53/Arf and IFN pathways and the mechanism of cell death. Since human melanoma cell lines are known to harbor either wild-type or mutant p53, we would also have the opportunity to explore the specific role of p53 in our gene transfer approach. For this, an additional set of vectors encoding human cDNA, with expression under the control of the constitutive CMV promoter, will be required.

The new vector constructs encoding human cDNA with either constitutive or p53-dependent expression will enable a deeper investigation of the molecular basis of the approach, including their behavior in cell culture and immunodeficient animals, the study of the contribution of p53 to the cellular response, and, possibly, their combined use with pharmacotherapeutic approaches.

### Seeking clinical relevance: use of patient-derived tumor samples

For decades, the number of established cell lines from tumor tissues has sharply increased, and this has undoubtedly resulted in many advances in the understanding of oncogenic mechanisms and the discovery of new targets for therapeutic interventions 58,59. As mentioned above, our own work has frequently employed established cell lines. During the isolation of *ex vivo* immortalized cell lines from normal tissues, some homeostasis mechanisms, such as cell cycle control, need to be inhibited, either by mutations of key genes or addition of growth factors in the culture medium. In comparison, cell lines derived from tumor tissues already exhibit these mechanisms, although often in a deregulated fashion 60,61. In addition to being cultured *in vitro*, these cells can be inoculated into immunocompromised animals to generate xenografts, which may be either heterotopic or orthotopic, providing the opportunity to study these cells *in vivo* 62.

While models based on human cell lines in monolayer cultures and their corresponding xenografts are widely used in cancer research, many limitations exist regarding this approach. Human cancers are known to have extremely heterogeneous cell populations comprising both neoplastic cells and stromal cells that actively contribute to tumor progression 63-66. Additionally, samples derived from different patients have unique sets of mutations, leading to differences in several signaling pathways and individual series of carcinogenic events 67. The establishment and propagation of these cell lines over time imply that only certain subpopulations will be selected, and the resulting clonality may not be representative of the original tumor heterogeneity 67,68. Therefore, the number of effective therapeutic tools that emerge from studies using cultured patient-derived cells is still small 59,69.

In contrast, PDXs involve the implantation of tumor tissues from patients directly into immunocompromised recipient mice 70. Unlike cancer cell lines, PDX models do not require *in vitro* culture steps, thus avoiding the selection of distinct cell populations and the exclusion of stromal components. The *in situ* physiological conditions encountered by these grafted cells in host mice, such as oxygen pressure, nutrients and metabolites, are similar to those found at the site of the patient's primary tumor. Experimental data suggest that overall genomic and gene expression profiles of PDXs are representative of the patients of origin and are stable throughout sequential *in vivo* generations 71.

Taking this into account, the establishment of each PDX is individually performed, preserving intra- and intertumor heterogeneity and providing a clear advantage when used for oncologic drug discovery and preclinical development 72. Evidence in the literature indicates success in predicting personalized anticancer treatments previously investigated using PDX models 73-75. Through the PDX approach, mutations in melanoma have been mapped in more detail, which provides complementary diagnostic data with greater predictive power to indicate the therapeutic approach to be adopted 76,77.

Another important feature recapitulated by the PDX model is the maintenance of a three-dimensional (3D) arrangement. Cells grown on a flat surface differ greatly from their 3D counterparts *in vivo* because of reduced cell-cell and extracellular matrix-cell interactions, a limitation already encountered with the traditional model of cancer cell lines 78-80. However, the culture model in 3D environments represents an intermediate approach between traditional cell culture and animal models, since it largely recapitulates the architecture of tissues *in vivo* 81-83.

Given that samples from patients are generally scarce and difficult to obtain, the use of 3D culture models enable the use of traditional cancer cell lines, prior to PDX models, for the standardization of viral infection kinetics, pharmacokinetics, molecular biology, biochemistry and imaging techniques. In addition, 3D cultures are less costly and labor-intensive than animal studies and should be prioritized at a preliminary stage. Experimental data show that the pattern of gene expression, mRNA splicing, intracellular signaling, cytoskeletal organization and secretion profile of cells grown in 3D cultures more closely resemble what is observed *in vivo* than when grown in two-dimensional (2D) cultures 84-88.

Initially, 3D cell cultures were derived from breast development studies and breast cancer models 89. Later, these methods were adapted for a variety of cells derived from different organ systems 90. Melanoma spheroids grafted in collagen gel matrix reproduce the architecture of a tumor encountered *in vivo*, including a gradient of oxygen and nutrients and a hypoxic and necrotic central zone 90,91. In this model, tumor heterogeneity is recreated in a manner similar to that found in patients, where only cells growing at the periphery have differential activity of the extracellular signal-regulated kinase (ERK). Consequently, small-molecule inhibitors of the mitogen-activated protein kinase (MAPK) pathway do not affect cells in the core of the spheroid 92,93. This observation highlights important implications that must be considered for the translation of novel targets for future therapies, both in terms of molecular responses and biodistribution of therapeutic agents, including adenoviral vectors.

Another important consequence of cell culture in a 3D environment is the enrichment of ‘cancer stem cell' subpopulations 94. Cancer stem cells represent rare subpopulations that have important characteristics including the capacity for autorenewal, tumor initiation and increased resistance to chemotherapy. Evidence suggests that cancer stem cells are the main cell type responsible for metastasis and tumor repopulation after debulking in response to chemotherapy 95-98. In 3D models of melanoma, a valuable strategy for obtaining, and consequently studying, this subpopulation is to culture melanocytes over extended periods of time in modified culture media 99. The use of these strategies contributes to physiologically relevant findings and greater chances of success in clinical trials.

### *Ex vivo* models for the activation of human immune cells

Since our strategy relies on not only the induction of tumor cell death but also the potential modulation of the tumor microenvironment, especially the tumor immune environment, it is crucial to investigate this interaction. As long as we use syngeneic mouse models, such assays are readily available. However, the transition to human models, including established cell lines or primary tumor samples, gives rise to a critical issue when considering tumor-immune interactions. *In vivo* models where human cells are implanted in mice create a situation where immunodeficient animals are necessary to minimize species incompatibility as described above, although this also means that the immune response cannot be evaluated. While mouse models with a humanized immune system are available, they are quite complex and associated with limitations in the emulated immune response 100. Alternatively, *in vitro* models permit the evaluation of specific aspects of the interaction between human immune cells and human tumor cells that have undergone some manipulation, such as gene transfer. Although such *ex vivo* models still do not reproduce all of the facets of treating a patient, they can reveal important aspects of the impact of treatments on the microenvironment, thereby opening a window to visualize important steps of immune activation.

In terms of the cancer-immunity cycle, our approach targets the first step, that is, the killing of tumor cells while releasing antigens and critical factors that activate DCs. As described above, we can transfer p14ARF and hIFNβ cDNA to established cell lines or primary samples. To model DC activation, we isolate mononucleated cells from blood and culture them with GM-CSF and IL-4 to induce the differentiation of monocytes to DCs. At that point, it is possible to modulate the maturation of the DCs by co-culture with previously treated tumor cells. A mature DC is expected to express co-stimulatory CD80, CD86, CD83 and MHC-II molecules. The state of maturation is thought to be dependent on the kind, duration and intensity of stimuli given to differentiated DCs

In addition to the characterization of mature DCs based on their immunophenotype, it is crucial to assess their ability to activate naïve T cells, as DCs are known to induce the T cell activation, marked by increased proliferation and change in differentiation markers such as CD4, CD8, Foxp3, RORγt, GATA-3 and T-bet. The activated T cells can then be co-cultured with tumor cells in vitro to induce tumor cell cytolysis and T cell proliferation. Ultimately, these activated T cells can be transplanted into immunosuppressed tumor-bearing mice used to generate the mature DCs to enable the assessment of T cell homing to the tumor and the induction of tumor cell death. Thus, the cancer-immunity cycle can be explored, although some adaptations may be necessary.

A critical issue when using *ex vivo* models of DC and T cell activation is the donor source used to acquire these cells. To minimize immune differences, the tumor, DC and T cells should come from the same patient. Nevertheless, DCs from cancer patients have been shown to generate a T cell response associated with increased regulatory T (Treg) cells 101. Moreover, the fusion of DCs with cancer cells has been shown to be superior to simple mixing of DCs with tumor cells in a vaccine model 102. Thus, the experimental design may greatly influence the result obtained with respect to both DC and T cell activation and functional assessment.

### Beyond the mouse: preclinical testing of gene therapy in canine melanoma

Few animal models recapitulate the entire cancer developmental process, starting with benign neoplasms, progressing to primary tumors and giving rise to metastases. Most murine models of human cancer involve the investigation of certain aspects of the complex interactions among the tumor, host and therapeutic modalities. As described above, we face difficult choices between established mouse or human cell lines and patient-derived cells since each of these cells has a different genetic profile. In addition, we encounter a serious limitation when human cells are implanted in immunodeficient mice. However, the study of cancer in dogs represents an important strategy in translational oncology. Spontaneous cancer can develop in dogs due to their relatively long life expectancy and exposure to environmental conditions that are similar to those experienced by humans 103,104. Dogs also have a complex immune system, including mature immune development accompanied by typical Treg cell responses 105.

In dogs, melanoma is the malignancy most commonly found on the digits and in the oral cavity. Canine and human melanomas share histological and biological similarities, including cell morphology, disease progression and response to therapies. Similar to human melanomas, canine melanomas are chemoresistant tumors 106.

Although not completely understood, the pathogenesis of canine melanoma involves the loss of function of tumor suppressor proteins, such as PTEN and p16/INK4a, common alterations that contribute to the origin of this cancer in both dogs and humans 107. BRAF is mutated in more than 56% of human cutaneous melanoma 108, but low rates of mutations of this gene are described in canine 109 and human mucosal melanoma 110. Both canine and human mucosal melanoma show the activation of cancer-related signaling pathways, such as AKT and MAPK, and are sensitive to the inhibition of these pathways 111.

These biological similarities between canine and human tumors may be responsible for the observed concordance in treatment responses. In veterinary medicine, many of the chemotherapy protocols are based on those used to treat humans 112. Moreover, sequencing of the canine genome was recently completed, and assay reagents and platforms are now commercially available, creating an opportunity to investigate tumor biology and drug response in dogs 113. Therefore, spontaneously occurring tumors in dogs may be considered preclinical models.

Various clinical trials of cancer therapies performed in dogs complement the use of the typically used murine cancer models for the development of new treatments 105,114-116. For example, gene therapy using herpes simplex virus TK and canine IFNβ combined with a subcutaneously delivered cellular vaccine expressing human IL-2 and GM-CSF significantly prolonged disease-free and overall survival while maintaining the quality of life of dogs with melanoma 117. In another study, the administration of the adenovector CD40L in stage III canine oral melanoma resulted in complete remission with 2 intratumor injections before cytoreductive surgery 118.

For both veterinary patients and their owners, this is an opportunity to participate in clinical trials of experimental therapies. However, for humans, these trials in veterinary patients are considered preclinical tests. This situation is referred to as a co-clinical trial, where the same drug is tested in both human and canine patients who have the same tumor type or mutation spectrum 105. With regards to gene therapy, co-clinical trials provide a unique opportunity to reveal mechanisms of pathogenesis and to identify correlations between outcomes in canines and humans.

Clinical trials in pets must be performed with informed consent of their owners and must be approved by an accredited animal care institute. The scientific motivation and translational study must be balanced in relation to the ethical and clinical perceptions of animal care. In general, pet dogs are treated in studies with designs that are similar to those used in clinical trials in human. However, the historical conventions of phase I, II and III studies may be less rigid while focus is maintained on developing the technical and biological aspects of the treatment strategy 119.

In the future, it is reasonable to expect clinical researchers will view naturally occurring cancers in dogs and other animals as complementary models to the translational study of new therapies. This approach may provide early toxicity detection, optimization of clinical trial design, reduction of costs and improvement in the future care of both canine and human cancer patients.

### Potential combinatorial approaches involving the p19Arf + IFNβ immunotherapy

Even though the experimental models described above can provide an opportunity for us to investigate vectors encoding p14ARF and IFNβ under conditions that more closely resemble human cancer, it is reasonable to presume that future translational approaches will most likely be applied in combination with other treatment modalities, a practice well established in the clinic for chemo, radio and targeted therapies 120 and notably underscored by the combined use of CTLA-4 and PD-1 checkpoint blockers 121.

Indeed, in view of the synergistic results obtained with dual CTLA-4 and PD-1 blockade in melanoma, their combined use was rapidly approved by the FDA and has highlighted the capability of targeting multiple immune pathways to provide benefit for patients who otherwise would not respond to cancer immunotherapy 122. Combinatorial approaches are also expected to circumvent acquired immunological resistance mechanisms and fuel the field of immunotherapy to move steadily forward 122.

However, caution is warranted, as, along with improved therapeutic results, there is a price to pay when targeting multiple checkpoint modulators, with an increase in autoimmunity and a series of immune-related adverse events 123. Although in the case of p19Arf + IFNβ gene transfer, we have not observed any signs of an autoimmune response, such as vitiligo in the melanoma model, we cannot rule out the possibility that such responses may be generated. New therapies, including ours, will require careful optimization and development to avoid these toxicities while still inducing antitumor immunity.

In fact, combinatorial strategies employing current and novel immunotherapeutic modulators and their mechanisms of action are being intensively studied in both preclinical and clinical settings 122. Furthermore, regarding the potential combinatorial approaches for our vectors, the immunomodulatory functions exerted by IFNβ are likely a critical target to be exploited. For example, in melanoma, a type I IFN signature correlated with CD8^+^ T cell infiltration and IFNβ production by tumor-associated DCs has been shown to be critical for mediating antitumor immunity 124 This function may prove to be particularly useful for the so-called cold tumors, which, in response to β-catenin signaling, lack the production of CCL4 and fail to recruit CD103^+^ DCs and produce IFNβ within the tumor microenvironment and, consequently, are deprived of the CXCL9/10 chemokines that drive T cell influx 125. Accordingly, these noninflamed tumors represent a challenge that current immunotherapeutic strategies have not yet been able to successfully target. Moreover, type I IFNs are notorious for increasing the expression of MHC-I molecules on tumor cells 126. Therefore, if we were to consider the disruption in the MHC-I pathway as a hallmark of immune evasion and therapy inefficacy 127, the use of IFNβ, especially under conditions where its expression levels and dynamics are controlled, could render tumor cells visible to the immune system, avoiding primary resistance to subsequent combinatorial immunotherapies.

In addition to these immunomodulatory functions, type I IFNs can promote different inhibitory mechanisms to regulate the amplitude and duration of the response, including the production of the indoleamine 2,3-dioxygenase (IDO) enzyme and, of special interest to our group, the expression of PD-L1 in both tumors and tumor-infiltrating immune cells 28,126. Although these inhibitory pathways may seem as an impediment to our therapeutic approach at first, we hypothesize that they actually create a potential opportunity for us to combine checkpoint modulators with our treatment regimen and ensure effective targeting of the IFNβ-mediated immunity cycle.

Combinatorial approaches can also augment cell death or even modulate pathways involved in ICD, potentially circumventing intrinsic defects or genetic alterations that may affect the ability of treated cells to elicit optimal antitumor immunity upon p19Arf + IFNβ gene transfer. For example, the use of other ICD inducers, such as doxorubicin and mitoxantrone chemotherapy, could be an interesting approach to induce caspase 3 activity along with the p19Arf + IFNβ necroptotic cell death and to change a resistant/non-ICD scenario to a *bona fide* ICD or remediate the capability of some agents to trigger ER stress responses, autophagy-dependent accumulation of ATP and HMGB1 release from the nucleus 128.

Our gene transfer approach promotes cell death and immune stimulation, thus creating a variety of opportunities for us to explore and optimize various aspects, including the development of vaccination strategies and the direct application of our vectors into the tumor mass. Nevertheless, many key aspects remain to be investigated, most importantly, the use of patient-derived tumor and immune cells to validate the evidence gathered from mouse models and the exploration of tumor responses in alternative models, such as spontaneously arising cancers in dogs. In terms of technological development, the production of viruses of pharmaceutical quality, that is, by following good manufacturing practices (GMP), is a critical step that is not currently available in Brazil. Nonetheless, the know-how required to conduct preclinical studies and clinical trials is readily available in our community and may be further supported by international collaborations.

The translational road ahead of us is long but certainly exciting. The goal of translational medicine is to evaluate therapeutic strategies that are successfully developed in preclinical models in clinical trials performed in humans. In addition, although we envision it as a straightforward path, the translational road should be seen as a two way road, with exchange of information between the bench and bedside, and vice versa. In this regard, it is critical that a multidisciplinary team of basic and clinical scientists work together to ensure that a clinically relevant and viable approach is developed as we progress along the road of translational research.

## AUTHOR CONTRIBUTIONS

Strauss BE, Silva GR, Vieira IL, Cerqueira OL, Del Valle PR, Medrano RF and Mendonça SA wrote and reviewed the text. Medrano RF designed the figure.
